# Evaluation of a Text Messaging Intervention to Support Self-Management of Diabetes During Pregnancy Among Low-Income, Minority Women: Qualitative Study

**DOI:** 10.2196/17794

**Published:** 2020-08-10

**Authors:** Lynn Yee, Shaneah Taylor, Maria Young, Makayla Williams, Charlotte Niznik, Melissa Simon

**Affiliations:** 1 Northwestern University Chicago, IL United States

**Keywords:** gestational diabetes mellitus, type 2 diabetes mellitus, mobile health, text messaging, mobile phone, pregnancy

## Abstract

**Background:**

Given the growing burden of diabetes in underserved communities and the complexity of diabetes self-management during pregnancy, the development of interventions to support low-income pregnant women with diabetes is urgently needed.

**Objective:**

This study aims to develop and pilot test a theory-driven curriculum of SMS text messaging for diabetes support and education during pregnancy.

**Methods:**

This was a prospective pilot investigation of a novel SMS text messaging intervention offered to pregnant women with pregestational or gestational diabetes mellitus and publicly funded prenatal care. Prior work yielded a conceptual model of diabetes self-management barriers and support factors in this population, which was used to guide curriculum development along with health behavior theories. Participants received three supportive or educational one-way text messages per week during pregnancy. In-depth semistructured interviews were performed at study exit to solicit feedback on the program. Narrative data were analyzed using the constant comparative technique to identify themes and subthemes.

**Results:**

Participants (N=31 enrolled and n=26 completed both interviews) consistently reported that SMS text messaging provided enhanced motivation for diabetes self-care, reduced diabetes-related social isolation, increased perceived diabetes-associated knowledge, enhanced comfort with the health care team, and reduced logistical burdens of diabetes during pregnancy. Participants requested enhanced interactive and customizable features in future intervention iterations.

**Conclusions:**

Pregnant women with diabetes who were enrolled in this pilot study of an SMS text messaging curriculum for diabetes support described enhanced motivation, knowledge, and comfort with diabetes self-care activities as a result of the health education intervention. The next steps include enriching the interactive features of the intervention and investigating the effect of the intervention on perinatal outcomes.

## Introduction

Diabetes mellitus poses a significant health burden to pregnant women [1,2]. Its prevalence has rapidly risen alongside the obesity epidemic, and it disproportionately affects low-income and minority women [3,4]. Effective treatment reduces the risk of many adverse maternal and child health outcomes [5]. However, successful management of diabetes during pregnancy is challenging because of the complexity of self-management skills, advanced patient education requirements, and high-level patient engagement required for optimal glycemic control. Diabetes significantly amplifies the requirements for self-care beyond normal pregnancy; these requirements may be particularly burdensome among low-income and minority women, who face additional social and structural barriers. Additionally, as pregnancy is a window of opportunity for health optimization [6] because of enhanced motivation and health care access, it is particularly critical to engage women with diabetes in healthy self-care during pregnancy.

Technology support interventions for individuals with chronic diseases can improve knowledge, engagement, and self-management of health conditions [1,7]. Technology expansion has made mobile health (mHealth) interventions a promising avenue for health promotion [8], especially in diabetes [9-12]. Text messages, for example, can be motivators, information sources, cues to action, reminders, and sources of support [13]. Outside of pregnancy, mHealth use is associated with improvements in glycemic control, self-care behaviors, treatment adherence, engagement, and self-efficacy [14-20]. However, existing interventions are not generalizable to pregnant women given their distinctly different clinical circumstances [21-26]. Data suggest that emerging pregnancy-related mHealth interventions may positively affect women’s health attitudes and behaviors [27,28], but there is a gap in available evidence-based technologies to address the complex needs of pregnant women with diabetes, particularly for low-income women.

Thus, we initiated a multiphase project toward developing an intervention for pregnant women with diabetes. We previously developed a model of barriers and facilitators to diabetes self-management, which informed our development of a theory-driven curriculum of SMS text messaging for diabetes support and education during pregnancy. The objective was to evaluate user experiences with this intervention, including feasibility, acceptability, and areas for improvement. We hypothesized that the delivery of a comprehensive curriculum of supportive and educational text messages aimed at promoting health during pregnancy with diabetes would be feasible and positively received.

## Methods

### Study Overview and Inclusion

This is a pilot investigation of a novel text message–based support intervention called *Texting for Diabetes Success* (TDS). Eligible women were aged 18 years or older, were English speaking, had publicly funded prenatal care, and had type 2 diabetes mellitus or gestational diabetes mellitus (GDM). Women with both types of diabetes were included because our prior data suggested that both groups experienced similar burdens and similarly complex health management [25,26,29]. Similarly, women were eligible for inclusion regardless of treatment modality. All women were receiving care at an academic hospital-based clinic that provides prenatal care for low-income women with public insurance. Participants were required to have a mobile phone and a willingness to receive text messages. They were eligible for participation after 10 weeks of gestation, and those who entered or transferred into prenatal care past 30 weeks were excluded as they had limited time for exposure to TDS. All participants provided written informed consent.

### Theoretical Foundation

There were 3 learning and health behavior theories that were used to develop this intervention: the Cognitive Load Theory, the Health Belief Model, and the Theory of Self-Efficacy. Each is discussed herein. Prior work from our group has applied Cognitive Load Theory [30,31] to frame the demands of pregnant women’s diabetes burdens, yielding a model of barriers and facilitators. Cognitive load refers to a task’s cognitive demand [31,32] and suggests that individuals have limited information-processing capacity, particularly with complex tasks [30,33,34]. Barriers included diabetes novelty, treatment disbelief, social instability and lack of support, limited nutrition comprehension and self-efficacy, psychological stressors, and logistical burdens of disease management [25,26,35]. Facilitators of self-care included self-efficacy, external motivation, supportive social and physical environment, and ability to self-regulate [29,36,37]. These findings formed the foundation for developing the TDS curriculum. In addition, 2 health behavior theories were applied to TDS development, as prior data suggest that effective interventions require a theoretical framework to derive the greatest benefit [8,13]. The Health Belief Model explains individuals’ engagement in health behaviors [38]. The Theory of Self-Efficacy emphasizes one’s belief in their ability to achieve their goals; learning and decision-making burdens of diabetes management demand self-efficacy, an issue particularly salient in pregnancy because of the short timeline for behavioral change [39,40].

### Intervention Development and Structure

We worked with a multidisciplinary health professional team including obstetricians, Registered Dietitians, a Certified Diabetes Educator, an Advanced Practice Registered Nurse (Nurse Practitioner), and other clinic personnel to develop a comprehensive curriculum of >150 messages to be delivered via text message. Early versions of messages underwent cognitive testing with pregnant patients with both type 2 diabetes mellitus and GDM using *think alouds*, a commonly used approach that encourages participants to determine what specific words make them think or feel [41]. Messages were also iteratively reviewed with clinical providers.

Messages were refined based on early provider and patient feedback. Messages were then organized into a curriculum of 3 messages per week using 3 theory-based content categories: logistical support, motivation, and information and education. For example, informational messages provide content about healthy foods using the Health Belief Model, whereas the Theory of Self-Efficacy Commonly guides motivational messages. Logistical messages primarily applied Cognitive Load Theory and offered tactical support such as appointment reminders or tips for the management of diabetes. Messages were designed to contain tips, motivational statements, or reminders that address barriers and facilitators identified in preliminary data (Table 1). Each message consisted of 1 to 2 short sentences or phrases (<150 characters) written in low literacy (eighth-grade literacy level or less) level, nonslang language, and consistent with participant preferences.

**Table 1:** Texting for Diabetes Success example messages.

Barrier or facilitator addressed		Example text messages
**Barriers**		
	**Disease novelty^a^**	
		Taking care of diabetes reduces your chance of high blood pressure and preeclampsia (toxemia).
		Pregnancy hormones make your diabetes worse. Blame the placenta, then show it who's the boss!
	**Failure of outcome expectation^b^**	
		Don’t get frustrated! Stick with your diabetes plan over time to get the healthy results you want for you and your baby.
		Taking care of your diabetes during pregnancy gets you on track for life. Hard work now means a longer, healthier life
	**Social chaos**	
		We know you have so much to do today. First thing on the list is making sure you and the baby are healthy - take care of your diabetes!
		Do you feel like you have the support you need? A healthy support system will benefit you and baby! Talk to us about resources at clinic.
	**Nutrition comprehension and action^c^**	
		Craving something crunchy and sweet? Grab a small apple and small handful of nuts. Eat healthy to control your diabetes during pregnancy.
		Try buying frozen veggies over raw ones, they can be cheaper and last longer! Vegetables can help control your blood sugar.
	**Psychological stressors**	
		Sometimes women can experience stress from relationships in their lives. Talking about what's bothering you can be healthy for both you and baby. Tell us about it at clinic.
		Identify your emotions. Say, “I feel upset, I'm not hungry” instead of reaching for the snack.
	**Burden of disease management^d^**	
		Needles? Blood sugar checks? Ultrasounds? Too much to handle? Take it one day at a time, a healthy baby in the end will be your reward.
		You have an OB appointment at PAC tomorrow. Don't forget to come fasting and bring breakfast.
**Facilitators**		
	**Diabetes self-efficacy**	
		We know you can do this! You can beat your diabetes!
		Are you feeling confident about your diabetes? Great, you've earned it!
	**External motivation**	
		Having a hard time keeping up with your diabetes? Everything you're doing now helps your baby!
		Have kids at home? Your healthy behavior means you are a great role model.
	**Supportive environment^e^**	
		Make eating healthy a family affair! Involve the whole family with your healthy meal plan.
		Too hard to exercise near home? Try the park district or your neighborhood community center.
	**Positive self-regulation^f^**	
		Everything you do now helps you live a longer life and be there for your baby. Good job!
		Seeing target blood sugars? Good job, you're on the right track! If not, talk to us!

^a^Represents the concept that diabetes and/or pregnancy are new learning concepts for the individual.

^b^Represents the concept that individuals may not believe that their actions will lead to the desired outcome.

^c^Represents the concept that nutrition recommendations may be both challenging to understand and challenging to execute.

^d^Represents the concept that having diabetes during pregnancy places a substantial burden on the patient to organize and complete logistical activities, scheduling, monitoring, appointments, and other health-related tasks, above and beyond normal pregnancy.

^e^Represents the concept that individuals may have the support of other individuals (eg, family) or a supportive physical environment (eg, safe places to exercise).

^f^Represents an individual’s ability to be responsive to feedback or data and then make subsequent changes in their health behaviors.

The overall curriculum was organized into 3 phases: a *ramp-up* at initiation, a middle period of sustained messaging, and a *wind down* at the end of pregnancy. All women received the same initial and end phases when possible; the quantity of the curriculum received in the intervening time was based on each woman’s gestational age at entry. Acknowledging that women may have varying experiences with diabetes management based on their history, the ramp-up phase was intended to support the novel aspects of being pregnant with diabetes, regardless of prior experience, and provide pregnancy-specific motivation. No postpartum messages were provided. Messages were delivered via a web-based, one-way messaging system.

### Participant Interviews and Analysis

After enrollment, participants completed demographic surveys that included queries on SMS text messaging access. Surveys were followed by brief interviews regarding experience with pregnancy and diabetes diagnosis, expectations for diabetes self-management requirements and burdens, and experiences with mHealth. Women then went through the remainder of the pregnancy receiving TDS.

After delivery, women underwent a 30-60 min exit interview about pregnancy struggles and support systems, experience of diabetes, and feedback about TDS. Feedback on TDS from this exit interview is the focus of this analysis. Women were asked about technical challenges, perspectives on content, positive and negative program features, favorite messages, feedback on frequency and timing of messages, and how the messages affected diabetes self-management. Women were asked about how the program supported them, if they would recommend it, and potential areas for expansion or improvement.

All interview questions were open-ended with probes as needed. Women were encouraged to speak freely and were informed that they could decline to answer any questions and that their responses would not affect their medical care. All interviews were audio recorded and conducted by trained research staff. We aimed to recruit a minimum of 30 participants to gain adequate feedback to facilitate future programmatic improvements, with the final sample size and stopping point determined based on the achievement thematic saturation [25,26,29,41].

Interviews were transcribed verbatim by a study team member. Dedoose (Dedoose, LLC), a secure qualitative data analysis software, facilitated thematic analysis of the transcripts by 3 authors using the constant comparative method [42]. This analysis explores the themes regarding participant experience and perceived usefulness and applicability of TDS. Analysts initially chose transcript excerpts and performed open coding on the feedback themes. An initial codebook was established through exploratory analysis of all transcripts and was used by both analysts. Standardized operational code definitions were created via team discussions. Additional codes that emerged inductively during subsequent coding were added to the codebook. All codes were reassessed for effectiveness of capturing themes after initial coding; ineffective codes were removed or reclassified. Discrepancies between analysts in code applications were reviewed by the team and resolved via discussion. Interrater agreement was not calculated given the team-based iterative approach to analysis. This study was approved by the Northwestern University institutional review board.

## Results

### Participant Demographics and Text Messaging Access

**Figure 1:**
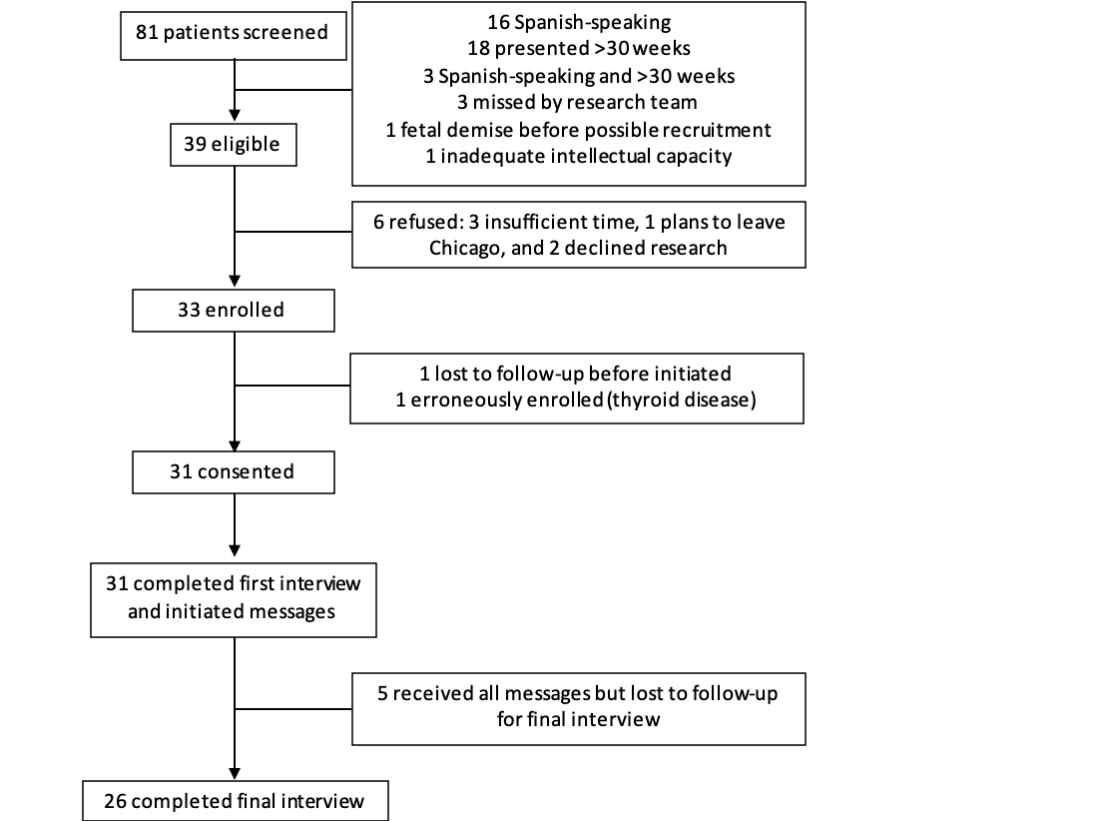
Participant flow.

Over an 8-month study period, 81 patients were seen in this practice and screened for eligibility, of whom 39 eligible women were approached for enrollment and 33 consented to participate. There were 6 women that declined participation because of insufficient time (n=3), plans to leave Chicago (n=1), or declining research participation (n=2). Of the 33 who consented, 1 was lost to follow-up (could not be reached) before the initiation of messaging and 1 was erroneously enrolled (did not have diabetes), leaving 31 participants who completed the first interview and received messages. Of these participants, 5 women were lost to follow-up before the final interview (could not be reached), leaving 26 participants who completed the exit interview (Figure 1). At the completion of 26 interviews, the team’s review of the data determined that thematic saturation had been achieved.

Participant demographics (Table 2) were representative of the clinic population. In this cohort of largely non-Hispanic black and Hispanic women, 100% (31/31) had publicly funded prenatal care and 71.0% (22/31) were high school graduates. The majority were multiparous and had pregestational diabetes. The majority (93.5%, 29/31) had a phone plan allowing unlimited text messages, and all had smartphones.

**Table 2:** Participant demographic characteristics.

Characteristics		Values
Age (years), median (IQR)		31 (27-34)
Public insurance, n (%)		31 (100)
**Race and ethnicity, n (%)**		
	Non-Hispanic Black	14 (45)
	Hispanic	16 (52)
	Asian	1 (3)
High school graduate or greater education, n (%)		22 (71)
**Employment, n (%)**		
	Full time for pay	5 (16)
	Part time for pay	7 (23)
	Homemaker or student	12 (39)
	Unemployed	7 (23)
Multiparous, n (%)		27 (87)
Married, n (%)		12 (39)
Pregestational diabetes, n (%)		21 (68)
Unlimited SMS text messaging plan, n (%)		29 (94)

### Participant Experiences of Texting for Diabetes Success

Interviews explored participant experiences with TDS and perceived usefulness. Emergent key themes included *increased connectedness, providing new information, support with logistical burdens, improving motivation,* and *receiving emotional support* (Table 3).

First, the theme of *increased connectedness* included reduced social isolation and perceived closeness with the health care team. Several participants introduced the idea of feeling connected to their health care providers through TDS; by receiving 3 messages per week, participants reported an enhanced sense of follow-up and individualized attention from their health care team. Participant 10 explained:

I actually loved it…every time…I would see the message it was like okay what is it today?

Similarly, TDS prompted participants to interact with their health care team, leading to a perceived stronger connection. Additionally, text messages seemed to reduce social isolation associated with diabetes; Participant 9 noted it was helpful “knowing someone cared.”

The second theme was of *providing new information*. Many participants reported that TDS helped them by providing information to better manage their diabetes. Participant 20 reported:

Some of the information was new, but some I already knew. But it’s not…hard to go over it again. Because a lot of us women that are diabetics, forget everything. You know and…me being 26 years old, we still don’t know everything about diabetes. It’s so much more out there, more to learn.

Similarly, Participant 25 stated:

Definitely…some of the messages encouraged me to look things online and just know a little bit more about the whole diabetes and being pregnant with it since there are so many risks with diabetes.

The third theme was *support with logistical burdens.* Participants nearly universally commented on logistical and appointment reminders as positive features. Participants perceived that TDS reduced logistical burdens associated with diabetes during pregnancy, including remembering frequent appointments (via appointment reminders) and support for other logistical burdens (such as reminders for eating, transportation, or childcare). Appointment reminder messages may have led to fewer missed appointments for some women. For example, Participant 8 reported:

Yeah that was good because actually was forgetting and did forget one time that I had an appointment thinking it was in two weeks, and it was actually that week and [the message] came right in time letting me know that my appointment was the next day.

The fourth theme was of *improving motivation*. Participants reported that the messages provided strong motivation to push through the challenges of managing diabetes and focus on self-care for the benefit of their fetus and themselves. Participant 21 explained:

It’s a way of motivation. To stay motivat[ed], you know help you understand it and give you ideas. And how to do reminders, you have [diabetes] but you can handle and take control of it.

Participants described how the messages motivated them not only toward positive behaviors but also away from negative behaviors. For example, participant 16 said:

Yeah, I mean they would stop me. Like the ones that would be like oh if you’re craving this, why don’t you eat an apple. And I was just like…how do they know! ...They would make me think before I did things or think like I wish I could be exercising right now.

The fifth theme was of *receiving emotional support*. As in our prior work [25], pregnant women with diabetes commonly report feeling socially isolated and emotionally stressed by the added burdens of a complicated pregnancy. Women in TDS reported that the connectivity of the program provided important emotional support to reduce stress, maintain positivity, and enhance self-efficacy. Participant 6 reported:

…you’re going through a lot of hormones when you’re pregnant. So it was like days I was feeling down. And I would just read the text messages and I would be like okay don’t give up, everything will be better. I don’t know, it was different [after receiving messages]

**Table 3:** Participant experiences of Texting for Diabetes Success.

Themes	Exemplary quotations
**Increased connectedness**	
	“Yeah, I did and I remember one time I received a text message and I think it was one had to do with the fruit. And I ended up eating something sweet and it didn’t affect my blood sugar, but I was like oh I wish I would have gotten my message a few minutes earlier because it would have stopped me*.*” (Participant 33)
	“Um I didn’t really use like I didn’t really use the recipes or anything like that, but I’ll say that it was a reminder like hey you know if I’m- They’re random so I could have been going to the store or something and buying something, hey I wanna buy that, but then I get a text message and I’m like oh maybe I shouldn’t be buying that you know, maybe I should go for a healthier choice because I have gestational diabetes and I shouldn’t be eating that. So I – I guess it poses as a reminder to do better.” (Participant 14)
**Providing new information**	
	“Ya know, I love the text messages. The text messages, they really help me, they really help me figure out a lot of things too and more about my diabetes. More of getting activity, getting rest, stuff like that I will always get a text message in the morning” (Participant 20)
	“I guess because when you’re in the situation of being diabetic you want to hear something you know funny or you know just to give a little fix to your little life there you know, it brings up…you know to do what you have to do and you know those type of sources [resources] will help a lot yeah.” (Participant 10)
**Support with logistical burdens**	
	“No I think it was pretty thorough but yeah the appointment reminders were very very good. I think it helps when you’re a mom already and you have things that you have to juggle around, the appointment reminders are helpful.” (Participant 13)
	“Thanks to the text messaging, I got reminders of my appointments to help me keep track, reminding me when to go and like what kind of ideas I could um be eating to stay away from the foods that are high in carbs.” (Participant 2)
**Improving motivation**	
	“That’s another support that you kind of have. Someone that’s not involved in your everyday life so to me it was very encouraging, you kind of have that little push.” (Participant 25)
	“I mean I think it helped with the motivation a lot. I mean you know the days when you’re like, hey I don’t want to do this or it’s ok to have, have a couple of pieces of this instead of just none or half to go back and look and ok no you need to take care of this. This is for you, this is for you child, it’s not for anyone else.” (Participant 21)
**Receiving emotional support**	
	“I think everything was perfect, because like I’m telling you I would forget sometimes to check my sugars and even sometimes when I was like getting stressed, those texts and someone would send me the text messages and they would lift me up.” (Participant 23)
	“I liked the…sometimes when I, when I was like tired or you know just sitting down and I received that text that was like a little extra push you know. If you feel stressed or go out and drink water or the changes of the recipes they were kind of fun.” (Participant 26)

### Recommending TDS

Women were asked to highlight positive programmatic feedback, including describing whether they would participate in TDS again or recommend it to a friend. Of the 26 women who participated in the exit interview, 23 would recommend TDS to a friend, whereas 2 would not, both of whom said they would recommend future more personalized versions. Participant 10 noted:

I would [recommend TDS] especially if she’s in the same situation. I mean it’s going to be stressful, it’s going to be hard, but I would recommend just because it teach you more resources apart of the clinic.

Similarly, participants reflected on the stresses of pregnancy and how their experiences with TDS influenced their self-care and attitudes toward the intervention. In reflecting on this experience, like many other participants, Participant 20 explained the multitude of reasons why she felt positively about her participation:

Every text message that you have gave me, sent to me, it was very good. There were no text messages that were boring. They were, they were getting to the point. They were understandable. And it was like a wakeup call. This is what you gotta do for your baby and yourself, to have a healthy baby, to have a healthy life. Ya know, I love the text messages…they really help me, they really help me figure out a lot of things too and more about my diabetes. More of getting activity, getting rest, stuff like that I will always get a text message in the morning. And be like ok this is my daily routine.

When asked about TDS drawbacks, some participants explained that they could not identify any. Participant 30 stated:

I mean it pretty much kept me aware of you know staying on track and it also alerted me to keep up with my doctor’s appointment…and it also gave helpful tips…Everything was helpful. There wasn’t anything negative stuck out to me with text messages or anything.

Participant 32 similarly summarized:

They would help you understand what to do and how to do it as far as what to eat and if you were to forget something or if you were to overeat or something how you would handle that. So it was very helpful.

### Areas for Improvement

Participants were encouraged to share areas of improvement to aid future development (Table 4). There were 4 major areas for expansion that were highlighted, including *individualized/customizable messages, interactive features, more frequent messages,* and *more recipes*.

The first theme was the desire for *individualized/customizable messages.* Women desired the program to reflect their names and other personal features. Others similarly desired that the program be tailored to personal psychosocial, medical, or logistical needs. Participant 4 shared:

If it was more individualized and I knew that I was receiving these text messaging for me personally for what I was going through and dealing with the diabetes then yes, it would have helped me more. Cuz then it would have prompted me to say, to read them, cuz I would say oh man okay you know this is something…I would have read it based on I knew it was for me.

The second area that was nearly universally highlighted was the desire for *interactive features.* Some women desired a feature that allowed for conversation with their health care team. Participant 20 elaborated:

It should be like chatting…If a diabetic don’t know what this is and she [texts] something, we can text her back what to do.

Other suggestions for an interactive platform were largely geared toward having user-friendly pictures, multimedia inclusion, or the ability to seek more information, suggestive of a smartphone app. For example, women desired pictures, links to resources, a library of resources, and a greater ability to access reliable content outside of the messages. Another interactive suggestion included the opportunity to share or read individualized stories of other pregnant women.

Third, women desired *more frequent messages*. Participant 14 shared:

I think it could be more, not annoying more but maybe like one more, just one in the beginning of the week and then one at the end of the week.

Participants commonly requested 4 to 5 messages per week, indicating that they were open to more frequent touch points for the motivational curriculum.

The fourth theme was the desire for *more recipes*. Women expressed that they would have appreciated more suggestions for snacks and recipes they could incorporate into their diets. Participant 3 stated:

I would like to have seen more recipe or…suggestions how to change because very often you’re used to having mashed potatoes and if you want mashed potatoes it’s better to have sweet potatoes or you know alternatives. That I would have liked to see more of that stuff.

**Table 4:** Texting for Diabetes Success areas for improvement.

Themes	Exemplary quotations
**Individualized or customizable messages**	
	“Now that would have been very much helpful. If it was more individualized and I knew that I was receiving these text messaging for me personally for what I was going through and dealing with the diabetes then yes, it would have helped me more.” (Participant 4)
	“Um during the week um or you know at the end of the week or at least once a weekend that will be very helpful to maintain those weekends because you know sometimes you have on your mind okay if I do really good during the week then you cheat on over the weekend” (Participant 12)
**Interactive features**	
	“That’s good because when you’re pregnant its hard, it’s very hard. Especially I mean, especially because of the baby you try to eat good you know but it’s still hard, but I think it just would work. I would put a bag of chips and then something nutritional next to it like kind of but yeah I think pictures would work out yeah, especially for an app.” (Participant 23)
	“I think uh support stories would be fine to go in there you know women who have experienced it and you know overcame it or women who still have diabetes after gestational.” (Participant 30)
**More frequent messages**	
	“Honestly I think that you should go up to five [messages]. ‘Cause once it get towards the end of the pregnancy, you need all the support you can get. Even if it’s from a text message that comes out of the blue like “hey, it’s almost over, we’re doing good, we can get through this.” Really you don’t know how much that really helped.” (Participant 28)
**More recipes**	
	“The messaging program helped a lot. I would recommend like a couple recipes like once a week or once a month. Hey here’s a great snack recipe or here’s a great lunch recipe or something” (Participant 21)

## Discussion

### Principal Findings

Diabetes during pregnancy is a major public health problem with important and long-lasting consequences for both women and their children. Innovative interventions to support pregnant women with diabetes self-management are lacking. Thus, we developed a theory-driven SMS text messaging program not only to provide psychosocial support but also to promote self-management skills and provide tactical support for pregnant women with diabetes. This intervention was feasible and well-received. Pregnant women with diabetes who were enrolled in this pilot study of an SMS text messaging curriculum for diabetes support described enhanced motivation and improved knowledge and comfort with diabetes self-care activities. Participants also had several suggestions for improvement, largely based on personalization and interactivity.

Although evidence-based mHealth interventions beyond general tracking and education provision for pregnant women are lacking, interest in such programs is high. The majority of pregnant women, including low-income and minority women, are interested in and have access to mHealth [43,44]. We found that access to this simple mHealth method—SMS text messaging—was high. Our findings mirror other reports on the growing interest in mHealth for pregnancy topics. Text4baby, for example, delivers health promotion messages to enhance general pregnancy behaviors and may positively affect health attitudes, although it is not specifically designed for women with diabetes [27,28,45,46]. GooDMomS is a web-based program that incorporates patient tracking, social networking, and weekly text messages and, in a small feasibility study, has shown promising preliminary results in assisting pregnant women with GDM manage diet and weight, although participants were largely nonminority and commercially insured women [47]. Related research has shown that culturally tailoring mHealth interventions to minority groups is vital to their success, thus underscoring the rationale for programs such as ours [48]. However, many programs still lack rigorous evidence-based or user-centered design [12], are designed for or studied among primarily nonminority women [47,49], are intended for general pregnancy support/tracking, or fail to address diabetes-specific needs [50,51]. Our findings point to the importance of addressing the myriad logistical, informational, social, psychological, and financial barriers to successful perinatal management of diabetes in the development of interventions [21-26].

Treatment of diabetes during pregnancy poses particularly complex challenges for low-income women. Multidisciplinary teams and treatment plans aim to optimize glycemic control and prevent complications via implementation of medical nutrition therapy, exercise, medication, and enhanced maternal-fetal surveillance [1,2]. Thus, perinatal care for diabetes necessitates advanced patient education and engagement along with communication, literacy, and organizational skills [25]. Such treatment requirements are particularly burdensome for women with a greater social disadvantage [21,22], who have multiple social and structural challenges to diabetes management [21-26,52]. Thus, an mHealth intervention that reduces these burdens may be particularly impactful and needed in this community. Ultimately, given the limited ability of in-person care to improve health behaviors, the implementation of mHealth interventions such as TDS may significantly enhance the care provided by the health care team [53]. Furthermore, when designed with both patient and clinician input to offer ongoing support beyond simply tracking glucose results, motivation-focused mHealth programs may be especially successful.

The next steps include enhancing the interactive features of TDS, scaling to a *high-tech* mHealth app, and investigating the effect of TDS on maternal and perinatal outcomes. Participants provided specific feedback about expansion opportunities. They were highly motivated for curriculum delivery via smartphone-driven technology, which they felt would best support their behaviors because of familiarity with other apps, the ability of an interactive and individualized app, and the desire to interact with an app on their own terms. Smartphone technology allows participants to interact with a technology-driven intervention in a nuanced, user-driven manner; for example, *favoriting* a message to view later is only possible with a smartphone. Smartphone architecture also allows the greatest flexibility for feature expansions; for example, merging an appointment reminder system, which was highly desired by participants, with an individual’s smartphone-based calendar may further enhance usability. Moreover, widespread smartphone availability, even in low-income communities, suggests that such an advancement may be widely accessible [43,54,55]. Advancement with such features may appeal to patients and enhance scalability for staff.

### Strengths and Limitations

A major strength of this study is the inclusion of a diverse group of participants who provided in-depth narrative perspectives. Furthermore, the intervention was developed with a robust theoretical underpinning and provided evidence-based, expert-driven content developed to meet the needs of the population of interest. Moreover, unlike interventions that primarily track glucose values, TDS included a curriculum for motivation and support. This approach is novel and supports the importance of patient-centered perspectives when developing health interventions.

However, there are several limitations to consider. Participants were primarily English-speaking women receiving care at a single academic medical center. Thus, as is common in qualitative research, findings are not widely generalizable, although they remain valuable for future intervention improvement. Second, although participants represented the demographics of the clinic population, participants may interact with mHealth differently than nonparticipants. Similarly, a small number of participants were unable to be reached for their exit interviews, and their experiences may have differed. Participants also included women with prior diabetes experience, including women with pregestational diabetes and women with gestational diabetes who had a history of gestational diabetes in prior pregnancies. Given the sample size, it was not possible to assess differential attitudes based on when diabetes was diagnosed or the type of diabetes diagnosis, although this topic is of critical importance for future intervention development. Future work may investigate differences in support needs and mHealth adoption based on diabetes type, prior experience with gestational diabetes, or other demographic characteristics, such as educational attainment. Finally, TDS was delivered without the ability to determine the proportion of messages received or read.

### Conclusions

In summary, pregnancy is a critical time period to improve women’s short- and long-term health, and improving support for women with diabetes has the potential to positively affect the health of families [6]. Pregnant women are motivated to improve health behaviors [56-58], and thus this period has the potential to spark lifelong behavior changes [59,60]. This pilot assessment of an SMS text messaging support program for pregnant women with diabetes demonstrates that offering such support to low-income women is desirable and feasible. Future areas of work include advancing the curriculum to meet the preferences and needs of this population and to promote its sustainability and scalability. Ultimately, the refinement, testing, dissemination, and implementation of interventions such as this may fill the gaps needed to positively influence women’s self-care behaviors and pregnancy outcomes.

## Abbreviations

GDM: gestational diabetes mellitus
mHealth: mobile health
TDS: Texting for Diabetes Success
